# Insulin Resistance and Environmental Pollutants: Experimental Evidence and Future Perspectives

**DOI:** 10.1289/ehp.1307082

**Published:** 2013-09-20

**Authors:** Tine L.M. Hectors, Caroline Vanparys, Luc F. Van Gaal, Philippe G. Jorens, Adrian Covaci, Ronny Blust

**Affiliations:** 1 Systemic Physiological and Ecotoxicological Research (SPHERE), Department of Biology, University of Antwerp, Antwerp, Belgium; 2 Department of Diabetology, Metabolism and Clinical Nutrition, and; 3 Department of Clinical Pharmacology/Toxicology, Antwerp University Hospital, University of Antwerp, Antwerp, Belgium; 4 Toxicological Center, Department of Pharmaceutical Sciences, University of Antwerp, Antwerp, Belgium

## Abstract

Background: The metabolic disruptor hypothesis postulates that environmental pollutants may be risk factors for metabolic diseases. Because insulin resistance is involved in most metabolic diseases and current health care prevention programs predominantly target insulin resistance or risk factors thereof, a critical analysis of the role of pollutants in insulin resistance might be important for future management of metabolic diseases.

Objectives: We aimed to critically review the available information linking pollutant exposure to insulin resistance and to open the discussion on future perspectives for metabolic disruptor identification and prioritization strategies.

Methods: We searched PubMed and Web of Science for experimental studies reporting on linkages between environmental pollutants and insulin resistance and identified a total of 23 studies as the prime literature.

Discussion: Recent studies specifically designed to investigate the effect of pollutants on insulin sensitivity show a potential causation of insulin resistance. Based on these studies, a summary of viable test systems and end points can be composed, allowing insight into what is missing and what is needed to create a standardized insulin resistance toxicity testing strategy.

Conclusions: It is clear that current research predominantly relies on top-down identification of insulin resistance–inducing metabolic disruptors and that the development of dedicated *in vitro* or *ex vivo* screens to allow animal sparing and time- and cost-effective bottom-up screening is a major future research need.

Citation: Hectors TL, Vanparys C, Van Gaal LF, Jorens PG, Covaci A, Blust R. 2013. Insulin resistance and environmental pollutants: experimental evidence and future perspectives. Environ Health Perspect 121:1273–1281; http://dx.doi.org/10.1289/ehp.1307082

## Introduction

The worldwide prevalence of metabolic diseases has substantially increased during the last few decades, and projections portend an even greater increase in the future (Finkelstein et al. 2012; Whiting et al. 2011). Although caloric consumption and sedentary lifestyle are surely major contributors to this rise, other nontraditional risk factors (e.g., environmental chemicals, stress, an altered gut microbiome) have been implicated as well (Thayer et al. 2012). The potential involvement of ubiquitous environmental pollutants in metabolic disease etiology, also known as the “metabolic disruptor hypothesis” (Casals-Casas and Desvergne 2011; Casals-Casas et al. 2008), has caught the interest of the scientific community and has been subject of intensive research over the last 5–10 years. At present, many institutions [e.g., U.S. National Institutes of Health (NIH 2011), U.S. National Institute of Diabetes and Digestive and Kidney Diseases (NIDDK 2011)] acknowledge and emphasize the need to understand the role of environmental exposures in metabolic disease development in order to inform future prevention and research strategies. Current prevention programs [e.g., U.S. Diabetes Prevention Program (Diabetes Prevention Program Research Group 2002), the European “IMAGE” program (Paulweber et al. 2010)] are aimed mainly at lifestyle interventions such as increasing physical activity and changing diet or, although less effective, pharmacological treatment (e.g., metformin, thiazolidinediones, orlistat). One of the major effects of these interventions and treatments is the management or improvement of insulin resistance (IR) (Nelson et al. 2013), a core pathophysiological process in the development of diabetes and a hallmark of most modern, metabolic diseases (e.g., metabolic syndrome, obesity, nonalcoholic fatty liver disease) (Samuel and Shulman 2012). Nevertheless, despite the central position of IR in metabolic diseases and current prevention strategies, the particular role of environmental chemicals in IR pathogenesis and the responsible molecular mechanisms have not been fully elucidated.

IR is defined as a state wherein normal concentrations of insulin evoke a less-than-normal biological response (Kahn 1978). It manifests itself in metabolically active tissues such as skeletal muscle, adipose tissue (peripheral IR), and liver (hepatic IR). Reduced sensitivity to insulin results in decreased insulin-stimulated glucose uptake together with a decline in glycogen synthesis in skeletal muscle and with impaired inhibition of lipolysis in adipose tissue. Hepatic IR is characterized by its selectivity: insulin fails to suppress glucose production, whereas fatty acid synthesis or lipogenesis is thought to remain intact or to be even hyperstimulated (Brown and Goldstein 2008). Thus, in the face of hyperinsulinemia in insulin-resistant conditions, the liver continues to produce glucose but also synthesizes large amounts of fatty acids and triglycerides, which accumulate in the liver, producing the pathological condition known as hepatic steatosis (Moon et al. 2012). Excess triglycerides are secreted via very low density lipoproteins, augmenting the levels of triglycerides in blood. The increased amount of fatty acids, derived from these triglycerides, may aggravate IR in muscle and adipose tissue and may contribute to β-cell dysfunction, ultimately leading to overt type 2 diabetes. As such, the triad of hypertriglyceridemia, hyperinsulinemia, and hyperglycemia characteristic for type 2 diabetes occurs (Brown and Goldstein 2008). Despite years of intensive research seeking to reveal the molecular pathogenesis underlying IR, the exact mechanisms are yet to be defined (Parker et al. 2011; Samuel and Shulman 2012).

To assess the relevance of the metabolic disruptor hypothesis for the human population, primary information can be derived from epidemiological studies. Given the increasing concern regarding the potential involvement of pollutants in metabolic disease etiology, the U.S. National Institute of Environmental Health Sciences/National Toxicology Program (NIEHS/NTP) (NTP 2011) organized a workshop in 2011 that resulted in the generation of a publicly accessible database containing > 200 human studies linking environmental pollutants to diabetes and obesity (NTP 2012). The role of pollutants in diabetes and obesity was compelling in the series of papers published following this workshop (Behl et al. 2013; Maull et al. 2012; Taylor et al. 2013; Thayer et al. 2012) as well as in other reviews (Alonso-Magdalena et al. 2011; Hatch et al. 2010; Hectors et al. 2011; Neel and Sargis 2011; Tang-Péronard et al. 2011). With regard to IR in particular, several studies have investigated the potential association between pollutants and markers of insulin sensitivity (e.g., Barregard et al. 2013; Chang et al. 2010, 2011; Chen et al. 2008; Dirinck et al. 2011; Færch et al. 2012; Kern et al. 2004; Lee et al. 2007, 2011; Nelson et al. 2010; Raafat et al. 2012; Wang et al. 2012). The most convincing evidence for a positive association of exposure to environmental pollutants and IR is with phthalates (James-Todd et al. 2012; Lind et al. 2012; Stahlhut et al. 2007) and air pollutants (Kelishadi et al. 2009; Kim and Hong 2012). Although these reports show a potential role for environmental pollutants in metabolic diseases, all studies emphasized the need for experimental evidence providing proof of causation of IR, diabetes, or obesity by pollutants and recommend the development of a standardized experimental testing strategy for this purpose (e.g., Taylor et al. 2013; Thayer et al. 2012).

Given that IR is a key feature of diabetes and most other metabolic diseases and that IR has a central position in current prevention strategies, the focus of the present review is on the role of environmental pollutants in IR pathogenesis. We aimed *a*) to summarize experimental studies linking pollutants to IR, and *b*) to gather information on available IR test models in order to discuss their suitability in IR toxicity testing. The latter is a first important step to streamlining future research on IR-inducing pollutants. In this regard, because metabolic disruptors are included in the group of endocrine-disrupting compounds (EDCs) (Casals-Casas and Desvergne 2011; Casals-Casas et al. 2008), much of the rationale behind EDC screening and toxicity testing also applies to metabolic disruptors. EDC screening is currently based on a tiered approach combining *in vitro* screening assays and both short-term and long-term *in vivo* assays [Organisation for Economic Co-operation and Development (OECD) 2012b; U.S. Environmental Protection Agency (EPA) 2013]. To suggest how future testing strategies to evaluate the relationship between metabolic disruptors and IR may look, we describe *in vivo* and *in vitro* end points that may be included in a comparable multilevel screening approach for the identification and prioritization of potential metabolic disruptors.

## Materials and Methods

We used Medical Subject Headings and keywords based on the search terms reported by Thayer et al. (2012) to screen the PubMed (http://www.ncbi.nlm.nih.gov/pubmed/) and Web of Science (http://images.webofknowledge.com/WOK45/help/WOS/h_database.html) databases to identify experimental studies relating IR to environmental pollution. One difference from Thayer et al. (2012) was the elimination of medical heading terms related to obesity and a specific focus on those with reference to IR. Studies reporting on developmental exposures and associations with the development of IR during adolescence or adulthood were not included. Throughout the literature on IR and environmental pollutants, a wide range of metabolic derangements related to IR are present [e.g., prediabetes, impaired glucose tolerance (IGT), impaired fasting glucose (IFG), insulin intolerance]. To avoid misinterpretation, we considered only studies specifically defining IR and discussing it as such. All retrieved studies (*n* = 23) are summarized in Supplemental Material, Table S1.

*State of the science—experimental studies linking pollutants to IR*. Experimental research currently available on the role of pollutants in the development of IR is summarized in Supplemental Material, Table S1. The reported effects on IR have been reviewed elsewhere for arsenic (Maull et al. 2012; Navas-Acien et al. 2006; Paul et al. 2007), dioxins (Remillard and Bunce 2002), and organophosphorus pesticides (Rahimi and Abdollahi 2007). In the studies included in the present review, the most compelling evidence for a potential link with IR pathophysiology is at hand for several persistent organic pollutants (POPs) (Hoppe and Carey 2007; Hsu et al. 2010; Ibrahim et al. 2011; Nishiumi et al. 2010; Ruzzin et al. 2010), phthalates (Rajesh et al. 2013; Srinivasan et al. 2011), bisphenol A (BPA) (Alonso-Magdalena et al. 2006; Batista et al. 2012), and air pollutants (Brook et al. 2013; Sun et al. 2009; Xu et al. 2011, 2012; Zheng et al. 2013). For phthalates and even more for air pollutants, epidemiological results (James-Todd et al. 2012; Kelishadi et al. 2009; Kim and Hong 2012; Lind et al. 2012; Stahlhut et al. 2007) have been confirmed by experimental research. Mice exposed to air pollution [in all studies tested as the particulate matter fraction with diameter < 2.5 μm (PM_2.5_)] showed either increased glucose intolerance [determined during intraperitoneal glucose tolerance tests (IPGTT)] (Sun et al. 2009; Xu et al. 2011; Zheng et al. 2013) or increased IR [based on homeostasis model assessment–IR (HOMA-IR), a validated IR index calculated by multiplying the fasting glucose with the fasting insulin level (Matthews et al. 1985)] (Brook et al. 2013; Sun et al. 2009; Xu et al. 2011, 2012; Zheng et al. 2013). To investigate the mechanisms underlying the observed IR, expression or phosphorylation status of different components of the insulin-signaling cascade was studied in aortic segments, liver, adipose tissue, or muscle (Sun et al. 2009; Xu et al. 2011; Zheng et al. 2013). The main conclusion of these studies was that air pollution seems to target all these tissues and the insulin receptor substrate 1/phosphatidylinositol 3-kinase/Akt (IRS-1/PI3K/Akt) signaling pathway, either by reducing the expression of related genes, or by reducing activating phosphorylation or inducing inactivating phosphorylation steps in this pathway. The most convincing proof of a direct role of PM_2.5_ in IR development can be found in a study by Brook et al. (2013) in which 25 human volunteers were transported for 5 consecutive days from a region with background levels of PM_2.5_ to a highly polluted area. Even for this relatively short exposure period, increased HOMA-IR, indicative for increased IR, was observed.

In opposition to the predominant single-compound studies listed in Supplemental Material Table S1, Ruzzin and colleagues tested relevant mixtures of POPs as they naturally occur in the food chain (in fish) (Ibrahim et al. 2011; Ruzzin et al. 2010). In their studies, rodents were fed high-fat diets containing POPs as part of fish oil (Ruzzin et al. 2010) and salmon fillets (Ibrahim et al. 2011). Chronic treatment resulted in severe impairment of whole-body insulin action, with both attenuation of insulin-stimulated glucose uptake in muscle and adipose tissue (peripheral IR) as well as reduced insulin-mediated suppression of hepatic glucose production (hepatic IR). These results were the first strong indications for a causal role of low-dose POPs in the development of IR. However, later reports from the same group showed some surprisingly opposite results (Ibrahim et al. 2012). Mice fed POP-containing whale meat showed improved insulin sensitivity and glucose tolerance compared with mice on isocaloric diets (Ibrahim et al. 2012). A proposed explanation is the dietary composition of the whale meat itself, which may counterbalance the potential negative health effects of POPs (Hennig et al. 2012; Ibrahim et al. 2012). Furthermore, in comparison with the salmon-based POP studies, the levels of certain POPs present in epididymal adipose tissue were 10–15 times higher when administered via whale meat (Ibrahim et al. 2011, 2012). Fried et al. (2010) showed that high concentrations of some POPs could improve hyperglycemia in a type 2 diabetic rat model exposed to high doses of dioxin (within 10-fold of a lethal dose). However, whether this hypoglycemic effect is directly caused by amelioration of IR or is the consequence of secondary effects due to other metabolic derangements [e.g., dioxins have been shown to inhibit gluconeogenesis directly (Zhang et al. 2012)] remains to be determined. Nevertheless, high doses of POPs may affect glucose homeostasis differently than low doses, urging in-depth dose–response characterization. The results of these studies highlight the complexity of interactions between environmental factors in the development of IR as dosing and the food matrix in which exposure occurs appear to greatly affect the outcome of a study.

Many more studies than those included in Supplemental Material, Table S1, have reported on a potential association between pollutants and altered insulin sensitivity, but did not specifically refer to the development of IR, or only indirectly showed the potential involvement of pollutants in IR pathophysiology. These associations are either based on *a*) altered glucose uptake by muscle or adipose tissue [e.g., bis(2-ethylhexyl) phthalate (DEHP) (Rajesh et al. 2013; Rengarajan et al. 2007); 2,3,7,8-tetrachlorodibenzo-*p*-dioxin (TCDD) (Kern et al. 2002); BPA (Sakurai et al. 2004); cadmium (Han et al. 2003)], *b*) changed expression of components of the insulin-signaling cascade [e.g., DEHP (Rengarajan et al. 2007)], *c*) altered expression of insulin-regulated genes and proteins [e.g., TCDD (Liu and Matsumura 1995); BPA (Sakurai et al. 2004); cadmium (Han et al. 2003)], or *d*) increased or decreased expression or synthesis of molecules which were previously causally related to IR. Examples for the latter are adipokines such as resistin [e.g., dichlorodiphenyldichloroethylene (DDE) (Howell and Mangum 2011)], adiponectin [e.g., polychlorinated biphenyl-77 (PCB-77) (Arsenescu et al. 2008), BPA (Ben-Jonathan et al. 2009)], and leptin [e.g., DDE (Howell and Mangum 2011)], and inflammatory mediators such as tumor necrosis factor α (TNF-α) [e.g., TCDD (Kern et al. 2002); BPA (Ben-Jonathan et al. 2009)], interleukin 6 (IL-6) [e.g., BPA (Ben-Jonathan et al. 2009)]. Although these studies were not included in the present review, they add to the knowledge base needed to assess the role of pollutants in IR specifically or metabolic diseases in general.

To summarize, most of the recent experimental studies that were intentionally designed to investigate pollutant effects on IR development (e.g., Batista et al. 2012; Lim et al. 2009; Ruzzin et al. 2010; Sargis et al. 2012) show convincing results and urge the need to accelerate and increase the efforts to investigate other ubiquitous pollutants within a uniformed testing scheme.

*A metabolic disruptor testing scheme: What do we have, and what are we heading for?* Lessons learned from EDC assays. Metabolic disruptors are a subset of EDCs (Casals-Casas and Desvergne 2011). As such, many of the necessary aspects included in EDC testing strategies to fully understand the mechanisms of action and effects of EDCs also apply to metabolic disruptors. For instance, many EDC effects describe nonmonotonic dose–response curves, occur at low doses (reviewed by Vandenberg et al. 2012), and are additive, synergystic, or antagonistic when considered in mixtures (Kortenkamp 2007). This kind of response is described for metabolic disruptors as well. A prime example is BPA, which exerts nonmonotonic and low-dose effects on the release of adiponectin from mature adipocytes (Hugo et al. 2008) as well as on the insulin content and concomitant secretion from pancreatic isolated islets (Alonso-Magdalena et al. 2008). Furthermore, Ruzzin et al. (2010) and Ibrahim et al. (2011) clearly showed that low-dose mixtures of POPs can induce IR, although in-depth knowledge of potential additive, synergistic, or antagonistic effects is, as far as we are aware, currently missing. Another previously encountered and much-debated issue with regard to EDC testing is the timing of exposure. In more classical EDC-oriented research (estrogenic, androgenic, and thyroid hormone disruption) exposure during critical developmental periods has been linked to altered reproductive function later in life (reviewed by Diamanti-Kandarakis et al. 2009) and even transgenerational effects (Anway and Skinner 2006; Walker and Gore 2011). Accordingly, recent studies have shown the potential of metabolic disruptors to “program” the development of obesity (reviewed by Janesick and Blumberg 2011) and IR or diabetes (Alonso-Magdalena et al. 2010) later in life following *in utero* or perinatal exposure, in some cases with lasting, transgenerational effects (Chamorro-García et al. 2013; Manikkam et al. 2013). Besides extensive dose–response testing; mixture evaluations for additive, synergistic, or antagonistic effects and effect assessment of exposures during sensitive life stages, different exposure lengths, sex-specific effects, differences in species and/or strain sensitivities have all been discussed for EDCs (Diamanti-Kandarakis et al. 2009; Kortenkamp 2007) and are to be considered when designing metabolic disruptor testing strategies. For EDCs, development and refining of such a testing strategy has gradually evolved and has recently resulted in the “OECD Conceptual Framework for the Screening and Testing of Endocrine Disrupting Chemicals” proposed by the OECD (2012b). This Conceptual Framework consists of standardized test guidelines to evaluate chemicals for endocrine disruption based on assays ranging from simple *in vitro* receptor binding assays, to physiological cellular assays, to whole-animal testing and even life cycle/multigenerational assays. Other EDC-screening programs also combine *in vitro* and *in vivo* assays to identify environmental chemicals with endocrine-disruptor capacities in a tiered testing strategy [e.g., Endocrine Disruptor Screening Program (U.S. EPA 2013)]. For metabolic disruptors, similar standardized testing schemes integrating *in vitro* and *in vivo* approaches are needed, but other end points than those currently present in EDC testing batteries are to be adopted.

One way to gain insight into the requirements for an IR toxicity testing strategy is to start from the assays and approaches that have been used for identification of IR-inducing chemicals. For that purpose, we assigned the currently used assays derived from Supplemental Material Table S1 to a level of toxicity testing based on acquired information and/or relevance of the end point (Table 1), keeping the OECD Conceptual Framework for EDC screening (OECD 2012b) in mind. Furthermore, we included in Table 1 some existing test methods in IR research that have not yet been implemented in the evaluation of pollutant effects on IR pathogenesis. Because this field of toxicology is new, *in vivo* testing will be important to provide evidence of causality; therefore, streamlining *in vivo* assays deserves primary attention. However, in the prospect of an IR toxicity testing strategy, the combination with mechanistic pathway-based screening assays using *ex vivo* or *in vitro* models becomes more evident, as discussed below.

**Table 1:** Summary of methods currently used to study pollutant effects on insulin sensitivity and suggested assays not currently adopted in IR toxicity testing.

End point	Method/models	Context/remarks	References^*a*^
L1 molecular event—*in vitro*/*ex vivo*
Insulin-signaling cascade (gene)	Real-time PCR, reverse transcriptase PCR, and gel electrophoresis: 3T3-L1 adipocyte cell line, primary adipocytes, dissected tissues (adipose tissue, liver, muscle)	Permanent change of the expression of genes in the insulin-signaling pathway may affect insulin sensitivity. Most commonly tested genes: *IRS*, *IRec*, and *GLUT4*.	Fang et al. 2012; Nishiumi et al. 2010; Rajesh et al. 2013; Sargis et al. 2012; Srinivasan et al. 2011
Insulin-signaling cascade (protein)	Western blot: L6 muscle cell line, 3T3-L1 adipocyte cell line, primary adipocytes, dissected tissues (aorta, adipose tissue, muscle, liver)	Most commonly used: pAkt/Akt, IRec or pIRec, IRS-1 or pIRS-1. Insulin stimulation necessary.	Batista et al. 2012; Fang et al. 2012; Ibrahim et al. 2011; Jubendradass et al. 2012; Lim et al. 2009; Nishiumi et al. 2010; Rajesh et al. 2013; Sargis et al. 2012; Srinivasan et al. 2011; Sun et al. 2009; Xu et al. 2011; Zheng et al. 2013
GLUT4 translocation	Separation of cytosolic and plasma membrane protein fractions (sucrose gradient or sonication), followed by Western blot analysis of GLUT4 protein content: 3T3-L1 adipocyte cells, dissected tissues (adipose tissue, skeletal muscle)	Insulin stimulation necessary.	Barnes and Kircher 2005; Rajesh et al. 2013; Srinivasan et al. 2011
Insulin-responsive genes^*b*^	Real-time PCR, reverse transcriptase PCR, and gel electrophoresis: *in vitro* models and *ex vivo* segments of adipose tissue, liver, and skeletal muscle	Insulin directly regulates expression of some genes. Examples of interesting targets: phosphoenolpyruvate carboxykinase (Logie et al. 2010); fatty acid synthase; sterol regulatory element-binding protein (Mounier and Posner 2006). Inability of insulin to stimulate/repress transcription of these genes may indicate IR. Insulin stimulation necessary.
L2 tissue-level response*—in vitro*/*ex vivo*
Glucose-stimulated insulin secretion	ELISA, RIA: isolated pancreatic islets	Chronic hyperinsulinemia may cause IR. For chronic exposures, insulin content may also be considered. May function as an indicator for indirect cause of IR.	Alonso-Magdalena et al. 2006; Batista et al. 2012
Glucose uptake	Addition of deoxyglucose followed by scintillation counting: 3T3-L1 adipocyte cell line, dissected tissues (adipose tissue, skeletal muscle)	Insulin stimulation necessary. Use of radiolabeled 2-deoxyglucose may affect the suitability of this assay in a screening context. Use of alternative approaches needs to be encouraged.	Barnes and Kircher 2005; Hsu et al. 2010; Ibrahim et al. 2011, 2012; Nishiumi et al. 2010; Rajesh et al. 2013; Ruzzin et al. 2010; Srinivasan et al. 2011
Adipokine and inflammatory cytokine production^*b*^	ELISA, RIA: 3T3-L1 cell line, primary adipocytes, dissected adipose tissue	Production of inflammatory cytokines such as TNF-α and IL-6 and some adipokines (e.g., resistin) is related to IR; others (e.g., adiponectin) are suggested to improve IR. Important species differences have been reported (Arner 2003). May function as an indicator for indirect cause of IR. Some pollutants (e.g., TCDD, DDE, PCB-77, BPA) affect the production of these molecules (Arsenescu et al. 2008; Ben-Jonathan et al. 2009; Howell and Mangum 2011; Kern et al. 2002).
Glucose production^*b*^	For methods, see de Raemy-Schenk et al. 2006; Foretz et al. 2010; Okamoto et al. 2009; Watts et al. 2005; Zhou et al. 2005: H4IIE cell line, HepG2 cell line, primary hepatocytes, liver slices, dissected liver	To test for hepatic IR, assays can be used in which liver cells are stimulated to produce glucose (e.g., dexamethasone stimulation), followed by insulin treatment. The degree of insulin sensitivity will determine the extent to which glucose production is reduced.
Glycogen synthesis^*b*^	Assessment of insulin-stimulated glycogen synthesis in liver and/or skeletal muscle: cell lines, primary hepatocytes, liver slices, dissected liver, dissected skeletal muscle	Insulin-stimulated glycogen synthesis can be assessed in combination with attenuation of insulin-inhibited glucose production (liver) or insulin-stimulated glucose uptake (skeletal muscle).
Lipolysis^*b*^	Assessment of insulin-mediated suppression of lipolysis in adipocytes: 3T3-L1 cells, primary adipocytes	Decreased insulin-inhibited lipolysis increases circulating free fatty acid concentrations that contribute to both peripheral and hepatic IR by impairing insulin-signaling pathways. In this way, induction of insulin IR in adipocytes may induce or aggravate IR in other tissues.
L3 organ-level response—*in vivo*
Glycogen content	Potassium hydroxide–based method followed by treatment with anthrone reagens or periodic acid–Schiff staining of glycogen: dissected liver, adipose tissue, and muscle		Fang et al. 2012; Rajesh et al. 2013; Zheng et al. 2013
Pancreatic β-cell function	ELISA or RIA: measurement of plasma insulin levels shortly (e.g., 15 min) after injection of glucose		Ibrahim et al. 2011, 2012
Skeletal muscle insulin sensitivity	Addition of glucose tracer during hyperinsulinemic–­euglycemic clamp to calculate glucose disposal		Ruzzin et al. 2010
Hepatic insulin sensitivity	Addition of glucose tracer during hyperinsulinemic–­euglycemic clamp to calculate hepatic glucose production or pyruvate tolerance test		Batista et al. 2012; Ruzzin et al. 2010
Adipose tissue insulin sensitivity^*b*^	Fatty acid tracer addition during hyperinsulinemic–­euglycemic clamp	Addition of fatty acid tracers allows monitoring of changes in lipolysis.
L4 whole-organism response—*in vivo*
Whole-body insulin sensitivity	Hyperinsulinemic–­euglycemic clamp	Alternatives: GTT + ITT. HOMA-IR is first line indication of IR, but can not be used on a stand-alone basis.	Alonso-Magdalena et al. 2006; Batista et al. 2012; Ibrahim et al. 2011; Lim et al. 2009; Ruzzin et al. 2010
Abbreviations: GLUT4, glucose transporter 4; IRec, insulin receptor; ITT, insulin tolerance test; L, level; p, phosphorylated; PCR, polymerase chain reaction; RIA, radioimmunoassay. L1 and L2 end points were tested with *in vitro* or *ex vivo* assays; L3 and L4 end points with *in vivo* assays. ^***a***^Only references that specifically investigated the role of pollutants in IR and used the corresponding end points to do so are included (for more information, see Supplemental Material, Table S1). ^***b***^End points have not yet been adopted in IR toxicity testing.			

*In vivo* testing of metabolic disruptors—top-down approach. A uniform *in vivo* testing scheme in which all pollutants are tested similarly is necessary for identification and potency characterization of pollutants that may pose increased risk for IR development. In proposing such a streamlined testing scheme, previous studies can be used as a roadmap for the dos and don’ts for future IR-pollutant research. In general, most of the studies first evaluated the presence of IR at the organism level (level 4) and then continued with in-depth analyses at the organ or tissue level to provide a more physiologic or even mechanistic basis for the observed effect (altered insulin sensitivity) (level 2 or level 1 assay), representing a top-down approach.

To determine IR at the organism level (level 4; Table 1), the hyperinsulinemic–euglycemic clamp technology is generally considered to be the gold standard (Mather 2009; Muniyappa et al. 2008). Although some studies draw the conclusion on induction of IR after pollutant exposure on this technique (Lim et al. 2009; Ruzzin et al. 2010), most studies use alternative measures. For instance, glucose tolerance tests (GTTs; oral, intravenous, or intraperitoneal) [e.g., Alonso-Magdalena et al. 2006; Batista et al. 2012; Khalil et al. 2010; Palacios et al. 2012 (see Supplemental Material, Table S1)] or HOMA-IR [e.g., Palacios et al. 2012; Ruzzin et al. 2010; Sun et al. 2009 (see Supplemental Material, Table S1)] were frequently performed or calculated to determine insulin sensitivity. However, when used alone, GTTs are usually considered a measure of glucose (in)tolerance, more than a measure of insulin (in)sensitivity (Muniyappa et al. 2008). Furthermore, the determination of IR based on surrogate indexes such as HOMA-IR, integrating fasting plasma glucose (FPG), and fasting plasma insulin (FPI) levels should be considered to be an initial indication of changes in insulin sensitivity but cannot be used to evaluate the potential of a pollutant to induce IR (Muniyappa et al. 2008). If used as an indicator, HOMA-IR calculation should incorporate species-specific adjustments to avoid erroneous interpretations as discussed by Mather (2009). Thus, standardization of the method used to determine the degree of insulin sensitivity is a precondition to allow testing pollutant effects on IR. Because hyperinsulinemic–euglycemic clamping is not easy to deal with in terms of animal handling, and is time and labor intensive, GTTs (preferably intraperitoneal or intravenous) combined with insulin tolerance tests (ITTs) are an advisable alternative for exploring pollutant effects on insulin sensitivity.

When IR is diagnosed using these techniques, more in-depth information is needed to evaluate the main impact and direct role of the pollutant in IR development. This is represented by the test methods of levels 2 and 3 (Table 1). One previously implemented approach exists in using glucose tracers to assess insulin-mediated suppression of glucose production (liver) or stimulation of glucose uptake (skeletal muscle) *in vivo* (e.g., Ruzzin et al. 2010). Accordingly, fatty acid or glycerol traces may be useful in assessing insulin-mediated suppression of lipolysis to determine adipose tissue IR although not previously applied in a toxicity testing context (Jensen and Nielsen 2007; Stumvoll et al. 2001). Besides these tracer experiments, the pyruvate tolerance test has also been used to determine hepatic IR (Batista et al. 2012).

Further support comes from *ex vivo* or *in vitro* testing of the degree of insulin-stimulated glucose uptake in isolated muscle and adipose tissue and insulin-suppressed glucose production in liver to elucidate whether either peripheral or hepatic insulin sensitivity is targeted (level 2; Table 1). In this regard, insulin-stimulated glucose uptake assays both in adipose tissue (primary adipocytes or 3T3-L1 cell line) and skeletal muscle (*ex vivo* testing on excised skeletal muscle segments) are quite popular (Barnes and Kircher 2005; Hsu et al. 2010; Ibrahim et al. 2011; Nishiumi et al. 2010; Ruzzin et al. 2010; Srinivasan et al. 2011), whereas assessing hepatic IR via *in vitro* or *ex vivo* glucose production assays has not been included in research on pollutant-induced IR yet. This is quite surprising because many studies have reported *in vitro* or *ex vivo* evaluation of hepatic insulin responsiveness based on the measurement of glucose production (e.g., de Raemy-Schenk et al. 2006; Foretz et al. 2010; Okamoto et al. 2009; Watts et al. 2005; Zhou et al. 2005). Because HOMA-IR is considered to predominantly indicate hepatic IR (Muniyappa et al. 2008), POPs (Ruzzin et al. 2010), particulate matter (Sun et al. 2009; Xu et al. 2011, 2012; Zheng et al. 2013), atrazine (Lim et al. 2009), and arsenic (reviewed by Maull et al. 2012; Palacios et al. 2012) may specifically target hepatic insulin sensitivity (see Supplemental Material, Table S1). As such, incorporation of a hepatic glucose production assay is crucial for assessing the physiologic mechanism underlying the observed systemic IR. Additional assays that may be performed in combination with these glucose production and glucose uptake assays are evaluation of insulin-stimulated glycogen production (mainly in liver and skeletal muscle) (e.g., Badin et al. 2011; Gao et al. 2010) or insulin-inhibited lipolysis (adipose tissue) (e.g., Lee and Fried 2012). These assays have also not been integrated in previous research on the impact of pollutants on insulin sensitivity.

In a final phase, some studies obtain mechanistic information (level 1; Table 1) by investigating the (gene or protein) expression and phosphorylation of key components of the insulin-signaling pathway (e.g., insulin receptor, components of the PI3K/Akt pathway, glucose transporter 4 expression and translocation), comparing insulin-stimulated and nonstimulated tissue fractions or isolated primary cells. The latter is important because although in some cases lowered expression of intermediates of this pathway could be related to reduced insulin signaling and thus might be involved in IR, a lack of insulin stimulation (e.g., Fang et al. 2012; Jubendradass et al. 2012; Zheng et al. 2013) does not provide solid and direct proof of decreased insulin sensitivity.

From top-down to bottom-up approaches: the need for mechanistic *in vitro* assays. At present, testing of pollutants to investigate their roles in IR is focused mainly at the organism level, followed by physiological or mechanistic evaluations at lower (organ, tissue, or cell) levels. The need for such evaluations is demonstrated by the fact that IR may be caused by the direct effects of a pollutant on insulin sensitivity or by its triggering of indirect mechanisms. Examples for the latter are increased synthesis and secretion of IR-inducing adipokines (e.g., resistin) (Howell and Mangum 2011) or inflammatory mediators (e.g., TNF-α, IL-6) (Ben-Jonathan et al. 2009; Kern et al. 2002) or hypersecretion of insulin (which may induce IR in the long term) (Alonso-Magdalena et al. 2006). As such, inclusion of the physiologic assays and mechanistic end points described above (e.g., glucose uptake, glucose production assays, expression of insulin-signaling cascade) together with assays that allow the monitoring of changes in adipokine and insulin secretion will improve our understanding of how pollutants can cause IR.

Besides knowledge on causality, pinpointing the mechanisms of metabolic disruption leading to IR will be very important in evaluating and developing dedicated mechanistic *in vitro* and *ex vivo* screens, making a bottom-up toxicity testing approach achievable in the near future. This bottom-up approach has not only a proven value in toxicity-testing strategies for mechanism-based hazard identification but its use is also inevitable when large numbers of pollutants need to be tested [Adler et al. 2011; Andersen and Krewski 2009; National Research Council (NRC) 2007b]: It allows rapid identification of potentially harmful pollutants, is cost-effective, and reduces the number of animals needed to establish a first indication for the potential risk of IR development [Interagency Coordinating Committee on the Validation of Alternative Methods (ICCVAM) 2004; Russell and Burch 1959].

In general, two types of *in vitro* assays are integrated in bottom-up toxicity testing strategies (Dix et al. 2007; Shukla et al. 2010): *a*) target-based screens or single–end point assays, which are used to investigate specific interactions with one defined target (e.g., receptor-binding, lactate dehydrogenase leakage); and *b*) cellular pathway–based assays in which toxicity pathways with adverse health events are modeled and perturbations of these pathways in response to a chemical can be measured.

Recent efforts to define and integrate target-based *in vitro* screens for metabolic disruptors emerged from an OECD report of the *Joint Meeting of the Chemicals Committee and the Working Party on Chemicals, Pesticides and Biotechnology* (OECD 2012a). In this OECD review, increased awareness was expressed that current EDC test guidelines do not test for all aspects of endocrine disruption. The report reviewed and described some new assays or novel end points to be incorporated in existing assays that would expand the repertoire of endocrine signaling pathways with pathways suggested to be involved in metabolic diseases, neurodevelopmental abnormalities, etc. For obesity, diabetes, and metabolic syndrome, the main suggested endocrine pathways are retinoid-X-receptor (RXR) and peroxisome proliferator–activated receptor (PPAR) signaling, with RXR and PPAR transactivation assays suggested to be the major mechanistic anchors or target-based screens (OECD 2012a). Indeed, the adverse obesogenic effect initiated by PPARγ activation is relatively well described (Casals-Casas et al. 2008; Grün and Blumberg 2007), and for each of the different levels of the tiered OECD conceptual framework, new assays or modified existing test guidelines have been suggested (e.g., 3T3-L1 differentiation as a level 3 end point, weight gain as a level 5 end point). In addition, for IR, pollutants that target PPARγ are interesting candidates for in-depth analyses because PPARγ agonists (thiazolidinediones) are used to treat this condition (Cariou et al. 2012). Similarly, glucocorticoid receptor activation, another metabolic nuclear receptor recently proposed for integration in EDC testing frameworks (OECD 2012a), may be a valuable single–end point screen because stimulation of this pathway is known to induce IR (Qi and Rodrigues 2007). Nevertheless, a mechanistic link between pollutant-induced PPARγ or glucocorticoid receptor activation or antagonism and the development of IR has not been identified in previous studies, although interactions have been suggested for TCDD with PPARγ (Remillard and Bunce 2002). This lack of knowledge of pollutant-specific mechanisms of action, limits, for now, the utility of these new metabolic nuclear receptor assays to identify potential IR metabolic disruptors. An alternative single–end point screen might include assays that allow the detection of changes in insulin-regulated gene expression. Interesting candidate genes in this regard are phosphoenolpyruvate carboxykinase (PEPCK), fatty acid synthase, and sterol regulatory element–binding protein (Mounier and Posner 2006), which are all directly transcriptionally regulated by insulin and are involved in key metabolic processes. The utility of PEPCK-based screening, for instance, has been proven by Logie et al. (2010), who developed a cellular IR model and defined IR solely on insulin-regulated PEPCK expression. Although single-gene expression assays might be a promising alternative in delivering a first indication, as for all single–end point assays, they are often too simplistic. Therefore, in general these single–end point-based assays are not ideal for identifying and screening metabolic disruptors if used on a stand-alone basis.

In our opinion, the identification of IR-metabolic disruptors is more achievable in the near future using the pathway-based approach, in line with the ongoing shift in toxicity testing strategies from traditional adverse effect–based screening toward mechanism-based testing (NRC 2007b). In this approach, pathways of toxicity (PoTs) are central, defined as cellular response pathways that, when sufficiently perturbed by an environmental agent, are expected to result in adverse health effects (NRC 2007b).

**Figure 1:**
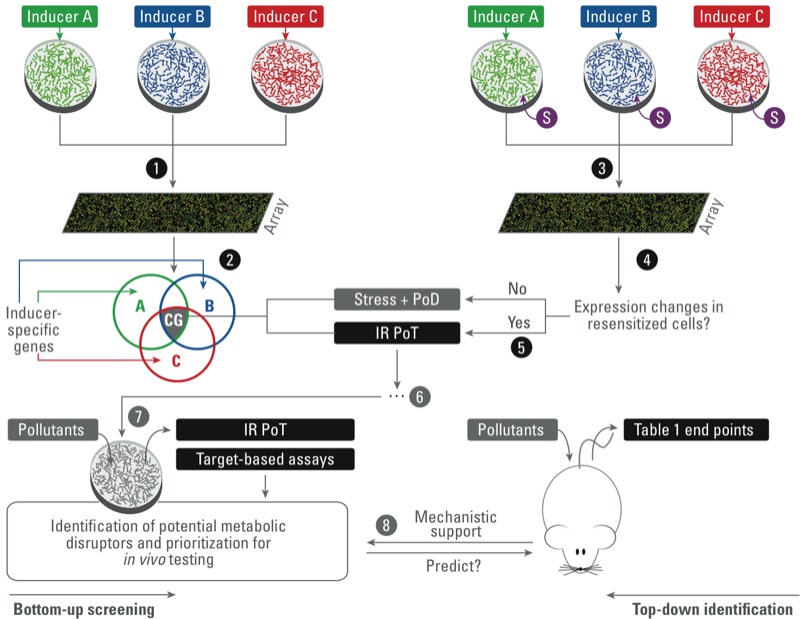
Schematic representation of an example of how an insulin-resistance pathway of toxicity (IR PoT) may be obtained. Steps 1–2: Exposure of in vitro models to three different inducers of IR followed by transcriptome analysis is expected to result in overlapping toxicogenomic profiles with “common genes” (CG) among the IR subtypes. This group of common genes is suggested to contain transcripts that are related to stress responses, to pathways of defense (PoD), as well as to the IR PoT. Steps 3–5: To separate the IR PoT genes, insulin-resistant cells may be treated with a sensitizer mix (S) containing drugs that improve insulin sensitivity. Transcriptome analysis of resensitized cells is expected to reveal which of the common genes among the IR subtypes expression is changed by resensitization. Those genes may then represent or define insulin sensitivity/resistance and, as such, reflect the IR PoT. Step 6: Further evaluation and validation steps are needed to assess how representative the IR PoT is and whether it is able to predict potential adverse *in vivo* effects. Steps 7–8: When IR PoT-based cellular assays can be developed, they should be integrated in a conceptual framework such as suggested in Table 1. Combined with single–end point or target-based assays, PoT-based cellular assays could be used as a mechanistic basis to identify and prioritize potential metabolic disruptors for further in-depth *in vivo* analysis.

In the development of screening systems for metabolic disruptors in a PoT-based toxicity testing approach, the combination of relevant *in vitro* models of insulin-sensitive tissues (liver, skeletal muscle, adipose tissue) with an omics approach may be a step ahead (Corvi et al. 2006; NRC 2007a). An example of how IR PoTs may be developed is shown in Figure 1. As described above, IR is a multifactorial disease, implying that different mechanisms lead to IR development. Moreover, at present, no explicit reference metabolic disruptors have been identified that induce IR. Therefore, it might be interesting to develop an assay looking at a robust end point that is reflective of IR but independent of the mechanism of the inducing factor. One way to obtain such an end point is the generation of a general IR PoT, based on transcriptome profiles from cells in which IR was induced with multiple factors (e.g., inflammatory factors, inducers of oxidative stress, glucocorticoids), representing the different IR-inducing mechanisms.

Imagine that inducers A, B, and C in Figure 1 render the cellular model insulin resistant via three different mechanisms. The resulting toxicogenomics analysis is expected to produce a set of overlapping genes common to all three inducers (segment CG in Figure 1, step 2), which is suspected to contain both general response genes (e.g., stress response pathways) and genes related to the pathway of defense (PoD; Hartung and McBride 2011) as well as those genes that are decisive in the development of IR (PoT). Separating these stress response and PoD genes from the core insulin-sensitivity determining genes requires an additional step. One plausible method, previously described by Hayward et al. (2011) and Konstantopoulos et al. (2011), would be to resensitize cells to insulin by exposing resistant cells to drugs commonly applied in the treatment of IR [e.g., biguanides (metformin), thiazolidinediones (pioglitazone), nonsteroidal anti-inflammatory drugs]. The strength of this approach lies within the coverage of the multifactorial nature of hepatic IR and selection of a common, inducer-aspecific PoT. Obviously, further validation of this gene set or PoT is needed before considering its potential application as a screening device for identifying IR-inducing pollutants.

## Conclusions

Overall, the role of pollutants in IR remains elusive. However, recent studies designed to investigate the impact of pollutants on IR development show a potential causative role, highlighting the need to accelerate and increase the efforts to investigate other ubiquitous pollutants with a uniform testing scheme. In suggesting such a testing scheme, a first important step was to extract a table of interesting test systems with indication of the respective end points currently used to study the effect of pollutants on IR pathogenesis. From the summary table, it is clear that most past, current, and ongoing test strategies in the field of IR toxicity testing use a top-down approach, starting at the organism level, followed by the evaluation of mechanistic end points at lower (organ, tissue, or cell) levels. The complexity of metabolic processes undeniably requires *in vivo* testing to assess the integrated response of whole-body energy homeostasis to pollutants. However, based on the rationale of EDC-screening frameworks, endeavors aiming at developing target- and pathway-based mechanistic *in vitro* assays should be encouraged to deliver mechanistic support for the observed metabolic disruption and allow cost- and time-efficient screening and identification of potential IR-inducing pollutants. Dedicated single–end point *in vitro* assays to detect obesogenic compounds have recently emerged. However, target-based assays for IR are missing, mostly because of the absence of a clear description of the molecular events preceding pollutant-induced IR development. Therefore, we hypothesize that, for now, the development of pathway-based *in vitro* screening approaches seems most feasible to allow mechanism-based identification and prioritization of potential IR-metabolic disruptors in the near future. With this review, we hope to emphasize the need for research on the link between pollutants and IR and to open thoughtful debate on how to generate a comprehensive testing strategy for metabolic disruptors.

## Supplemental Material

(782 KB) PDF
