# Supplementary material for: Insulin Resistance and Environmental Pollutants: Experimental Evidence and Future Perspectives
Source: Environ Health Perspect. 2013 Sep 20;121(11-12):1273–81. doi: 10.1289/ehp.1307082 (PMC3855520; doi:10.1289/ehp.1307082)
Supplement: (782 KB) PDF [file ehp.1307082.s001.508.pdf]

Environmental Health Perspectives doi:10.1289/ehp.1307082

Supplemental Material

Insulin Resistance and Environmental Pollutants: Experimental Evidence and Future Perspectives

Tine LM Hectors, Caroline Vanparys, Luc F Van Gaal, Philippe G Jorens, Adrian Covaci, and Ronny Blust

Table of Contents

Supplemental Material, Table S1. Overview of experimental studies investigating the link between pollutants and IR…………………………………………………...………..pages 2-10

References……………………………………………………………………….pages 11-13

Supplemental Material, Table S1[a,b]

Source	Chemical	Study	Dose and time	Ins	Endpoints	Observations suggested to be associated with IR	IR
Palacios et al. 2012	Arsenic (As) and lead (Pb)	Rat; *In vivo*	30 ppb As + 53 ppb Pb (contaminated drinking water from Antofagasta city); 3 months	No	FPG (=); FPI (↑); HOMA-IR (↑); OGTT: Glucose (↑), Insulin (↑)	Authors state that exposure to Pb and As contaminated drinking water induced IR in male rats. This conclusion was derived from calculation of HOMA-IR and confirmation by OGTT tests, in which especially male mice had higher glucose and insulin levels compared to the male control group.	WB
Lim et al. 2009	Atrazine	Rat; *In vivo*; *In vitro* (L6 cell line)	30 or 300 µg KD; 5 months (regular diet vs high fat diet); L6: 100 µg/mL; 24 – 48 h	Yes	FPG (↑); Plasma insulin (↑); HOMA-IR (↑); IVGTT: Glucose (↑), Insulin (↑); Hyperinsulinemic-euglycemic clamp: GIR (↓), Insulin sensitivity index (↓); Muscle (L6 cells) protein expression: pAkt/total Akt (↓)	IVGTT and hyperinsulinemic-euglycemic clamp technology showed IGT and whole body IR for animals exposed to 30 and 300 µg KD atrazine fed on a regular diet. The insulin sensitivity index was also decreased for both atrazine treatments. In L6 muscle cells, atrazine abolished insulin mediated Akt phosphorylation.	WB, P(M)
Khalil et al. 2010	Benzo[a]pyrene (BaP)	Mouse; *In vivo*	2.5 mg/kg bw; 5 weeks; Mice were fed normal diet, high-fat diet (HFD) and HFD supplemented with BaP.	No	Plasma insulin (↓); IPGTT (glucose) (=)	Khalil et al. (2010) report slightly decreased plasma insulin levels in HFD + BaP, while no further worsening of glucose intolerance was observed compared to HFD alone. Decreased expression of glucagon-like peptide 1, an incretin which stimulates insulin secretion, in BaP-treated mice was proposed to explain decreased insulin levels. Furthermore, BaP exacerbated HFD-induced inflammatory gene expression and expression of genes related type 2 diabetes in bowel and/or liver. As such, it was concluded that BaP might increase the risk of type 2 diabetes without worsening IR.	WB
Alonso-Magdalena et al. 2006	Bisphenol A (BPA)	Mouse; *In vivo*; *Ex vivo* (glucose stimulated insulin secretion from isolated pancreatic islets)	100 µg KD; 4 days	No	Plasma glucose (=); Plasma insulin (↑); IPGTT (glucose) (↑); IPITT (glucose) (↑); Glucose stimulated insulin secretion (insulin) (↑)	BPA-treated fed mice showed hyperinsulinemia and unchanged blood glucose concentrations, a symptom of IR. Further evidence was obtained in IPGTT and ITT tests, where IGT and insulin intolerance were observed in BPA-exposed mice. BPA was thus implied as potential IR-inducing factor.	WB

Source	Chemical	Study	Dose and time	Ins	Endpoints	Observations suggested to be associated with IR	IR
Batista et al. 2012	Bisphenol A (BPA)	Mouse; *In vivo*; *Ex vivo* (glucose stimulated insulin secretion from isolated pancreatic islets)	100 µg KD; 8 days	Yes	Plasma glucose (fed) (↓); Plasma insulin (fed) (↑); IPGTT (glucose) (=); IPITT (glucose) (↑); Glucose stimulated insulin secretion (insulin) (↑); Muscle protein expression: IRS-1 (↑), PI3K (p85) (=), Akt (=), pIRβ (↓), pAkt (Ser^{473}) (=), pAkt (Thr^{308}) (↓), pErk (↓); Liver protein expression: IRS-1 (↑), PI3K (p85) (=), Akt (=), pIRβ (↓), pAkt (Ser^{473}) (=), pAkt (Thr^{308}) (=), pErk (=); Pyruvate tolerance test (liver; gluconeogenesis) (=)	Treated mice showed reduced insulin sensitivity as determined with ITTs. In skeletal muscle of BPA-treated mice which were injected with insulin, reduced phosphorylation of the insulin receptor β-subunit and of Thr^{308} of the Akt protein, compared to vehicle-treated insulin exposed mice was observed. Furthermore, BPA treatment also seemed to affect the MAPK signaling pathway as illustrated by absence of insulin mediated ERK phosphorylation. In liver, impairment of the insulin signaling pathway by BPA could only be observed at the level of insulin stimulated tyrosine phosporylation of the insulin receptor β-subunit. No further effects in downstream insulin signaling pathways were observed, nor changes in MAPK signaling. A pyruvate tolerance test showed no effects on pyruvate induced gluconeogenesis. In both liver and muscle, increased IRS-1 expression was observed, suggested to be a counter regulatory mechanism to overcome IR. Based on these observations, the authors conclude that BPA induced IR is mainly due to targeting of skeletal muscle.	WB, P(M), H
Srinivasan et al. 2011	Bis(2-ethylhexyl) phthalate (DEHP)	Rat; *In vivo*; *Ex vivo* (insulin stimulated glucose uptake in skeletal muscle segments)	10 or 100 mg KD; 30 days	Yes	FPG (↑); Plasma insulin (↓); OGTT (glucose) (↑); Muscle gene expression: IRec (↑), IRS-1 (↑), GLUT4 (=); Muscle protein expression: IRec (↓), IRS-1 (↓), Akt (↓), GLUT-4 (cytosol) (↑), GLUT-4 (membrane) (↓), pIRS-1 (↓), pAkt (Ser^{473}) (↓); Glucose uptake (muscle) (↓)	100 mg KD DEHP increased FPG and decreased plasma insulin levels. Furthermore IGT was observed and insulin stimulated glucose uptake in muscle of exposed rats was decreased. In muscle, changes in IRec, IRS-1, Akt and GLUT4 expression (mRNA and/or protein) were reported and Akt phosphorylation status was reduced (for the highest DEHP dose). The authors conclude that their results provide experimental data that DEHP exposure causes impaired insulin signaling which leads to the onset of IR.	WB, P(M)

Source	Chemical	Study	Dose and time	Ins	Endpoints	Observations suggested to be associated with IR	IR
Rajesh et al. 2013	Bis(2-ethylhexyl) phthalate (DEHP)	Rat; *In vivo*; *Ex vivo* (insulin stimulated glucose uptake in adipose tissue)	10 or 100 mg KD; 30 days	Yes	FPG (↑); Adipose glycogen content (↓); Glucose uptake (adipose tissue) (↓); Glucose oxidation (adipose tissue) (↓); Adipose tissue gene expression: IRec (↓), IRS-1 (↓), GLUT4 (↑,=); Adipose tissue protein expression: IRec (membrane) (↓), IRS-1 (↓), pIRS-1 ($Ser^{636/639}$) (↓,↑), pIRS-1 (Tyr^{632}) (↓), β-arrestin2 (↓), Akt (=), pAkt (Ser^{473}) (↓), AS160 (↓), SREBP-1c (↓), GLUT4 (cytosol) (↓), GLUT4 (membrane) (↑), pGLUT4 (Ser^{488}) (↑)	Rajesh et al. (2012) conclude that DEHP treatment induces glucose intolerance, mediated by reduced GLUT4 protein expression and translocation, which diminishes glucose uptake and oxidation. DEHP is suggested to cause glucose intolerance by reducing expression of insulin signaling intermediates (IRS-1/Akt pathway) or by inhibiting phosphorylations which activate intermediates in the insulin signaling cascade. The mechanisms thought to be involved are increased production of ROS and lipid peroxidation. Based on these findings, the authors concluded that DEHP is associated with IR in adipose tissue.	WB, P(A)
Barnes and Kircher 2005	$HgCl_2$	Mouse; *In vitro* (3T3-L1 cell line)	0.5, 1, and 5 μM; 24 h	Yes	Glucose uptake (3T3-L1) (↓)	Mercury had no effect on the levels of insulin-mediated glucose transport, but since mercury itself increased glucose transport in 3T3-L1 cells, the authors concluded that there was a decrease in the insulin-mediated component of glucose transport. The mechanism suggested to underlie the limited cellular insulin response, is induction of stress (increased p38 phosphorylation). Because $HgCl_2$ decreased insulin-mediated glucose transport, a characteristic of IR, the authors state that $HgCl_2$ might have an impact on glucose homeostasis.	P(A)
Mostafalou et al. 2012	Malathion	Rat; *In vivo*; *Ex vivo* (activity of liver enzymes)	25, 50 or 100 mg KD; 32 days	No	FPG (↑); OGTT (glucose) (↑); Enzyme activity (liver): PEPCK (↑), G6Pc (↑)	Malathion treatment (25 and 50 mg KD) causes hyperglycemia as determined by FPG measurement and dose-dependently induces glucose intolerance demonstrated by the OGTT test. The observed postprandial hyperglycemia was suggested to be partly mediated by increased PEPCK and G6Pc activity in malathion-treated rats. Since insulin normally inhibits activity of both enzymes, malathion toxicity was suggested to specifically target insulin signaling pathways. Furthermore, TNF-α, inflammatory pathways and oxidative stress were suggested as mediators of impaired insulin signaling in liver of malathion-treated rats and the induced (hepatic) IR.	WB, H

Source	Chemical	Study	Dose and time	Ins	Endpoints	Observations suggested to be associated with IR	IR
Jubendradass et al. 2012	Nonylphenol	Rat; *In vivo*	15, 150 or 1500 µg KD; 45 days	No	FPG (↑); FPI (↑); Liver protein expression: IRec (↓), IRS-1 (↓), IRS-2 (↓), PI3K (↓)	Nonylphenol increased FPG and FPI and decreased the expression of proteins involved in insulin signaling pathway in liver: IRec, IRS-1, IRS-2 and PI3K. Because long-term administration of nonylphenol caused hyperglycemia and hyperinsulinemia, the authors speculated that nonylphenol could induce an IR state in rats.	WB, H
Fang et al. 2012	Perfluoronona noic acid (PFNA)	Rat; *In vivo*	0.2, 1 or 5 mg KD; 14 days	No	FPG (↑); Liver glycogen content (↑); Liver gene expression: Glucokinase (↓), glucose-6-phosphatase (↑), glucose transporter 2 (↑), PI3Kca (↓); Liver protein expression: pIRS1 (↓), pPI3K (↓), pPDK1 (↓), pAKT (↓), pGSK3β (↑)	PFNA treatment increased FPG associated with decreased expression of PI3K/Akt pathway components in liver. Furthermore, glycogen content was increased as well as glucose-6-phosphatase gene expression. The authors suggest that by attenuation of the PI3K/Akt pathway PFNA may cause IR and may explain the observed increase in FPG.	H
Ruzzin et al. 2010[c]	Persistent organic pollutant (POP)-mixture	Rat, mouse; *In vivo*; *Ex vivo* (insulin stimulated glucose uptake in muscle and adipose tissue). *In vitro* (3T3-L1 cell line)	*In vivo*: 28 days of exposure through diet. Diet: control diet (C), high fat diet (HF), high fat diet with added crude salmon oil (HFC) or high fat diet with added refined salmon oil (HFR). Both crude and refined salmon oils were contaminated with a mixture of POPs as they naturally occur in fish oil. The POP levels were much higher in crude oil, compared to refined oil. 3T3-L1: POP mixtures in different concentrations, 48 h	Yes	Basal plasma insulin (↑); Basal plasma glucose (=); HOMA-IR (↑); Basal HGP (=); Hyperinsulinemic-euglycemic clamp: Rd (↓), GIR (↓), HGP (↑); Glucose uptake (adipose tissue) (↓); Glucose uptake (muscle) (↓); Glucose uptake (3T3-L1) (↓)	Hyperinsulinemic-euglycemic clamp showed that HFC (highest POP concentration) aggravated IR induced by HF diet, while no effects were seen for HFR diet. Hepatic IR was shown by reduced suppression of hepatic glucose production by insulin for HFC and HF diets, while also peripheral IR could be detected for the HFC diet. Peripheral IR was shown by decreased insulin stimulated glucose uptake in isolated muscle (HF and HFC) and primary adipocytes (HF, HFR, and worsened by HFC). Exposure of 3T3-L1 cells to POP mixtures also impaired insulin stimulated glucose uptake and mixtures of DDTs and PCBs had the strongest inhibitory effect on insulin action.	P(M), P(A), H

Source	Chemical	Study	Dose and time	Ins	Endpoints	Observations suggested to be associated with IR	IR
Ibrahim et al. 2011[c]	Persistent organic pollutant (POP)-mixture	Mouse; *In vivo*; *Ex vivo* (insulin stimulated glucose uptake in skeletal muscle); *In vitro* (3T3-L1 cell line)	*In vivo*: 8 weeks of exposure through diet. Diet: control diet, very high fat diet (VHF), very high fat diet containing farmed salmon fillet (VHF/S) or very high fat diet containing farmed salmon fillet with reduced POP concentrations (VHF/S_{-POPs}). Both salmon fillets contained POPs, but VHF/S_{-POPs} contained ≈ 50% lower POP concentrations compared to commercial farmed salmon fillet (VHF/S).	Yes	IPGTT (glucose) (↑); IPITT (glucose) (↑); Glucose stimulated insulin secretion (insulin) (↑); Basal plasma glucose (↑); Basal plasma insulin (↑); Glucose uptake (muscle) (↓); Muscle protein expression: pAkt/total Akt (↓)	Mice fed VHF diet were glucose intolerant, which was worsened by VHF/S. Furthermore, increased insulin production was observed in VHF/S-fed mice in response to a glucose challenge and insulin induced glucose clearance was reduced in VHF/S-fed relative to C-fed and VHF-fed mice, all features demonstrating a state of IR. *Ex vivo* evaluation of peripheral IR (muscle) showed that insulin stimulated glucose uptake was reduced in mice fed VHF and VHF/S compared to C-fed mice. In addition, insulin stimulated phosphorylation of Akt was decreased in skeletal muscle both for VHF and VHF/S fed mice. Reducing POP-levels in VHF/S diet, resulted, in general, to a better whole body insulin sensitivity (insulin tolerance test). Glucose levels were the same for both mice fed VHF/S as VHF/S_{-POPs} comparing FPG and GTT, while insulin plasma levels were decreased for VHF/S_{-POPs}– mice and glucose stimulated insulin production was also reduced.	WB, P(M)
			An alternative treatment consisted of exposure through diet for 6 weeks. Diets were composed of a control diet (chow low-fat diet), a western diet (WD) or WD containing farmed salmon fillet (WD/S).			*Ex vivo* muscle glucose uptake showed enhance insulin action in VHF/S_{-POPs}-fed mice. For the WD-experiment, it was shown that, in fed condition, mice fed WD/S had a mild increase of blood glucose and a dramatic increase in plasma insulin levels, suggesting whole body IR. GTT and ITT confirmed IGT and systemic IR. Insulin stimulated glucose uptake was also significantly reduced in WD/S-fed mice. All these results highlighted, according to the authors, that POPs may have a causal role in metabolic disorders.	
Ibrahim et al. 2012	Persistent organic pollutant (POP)-mixture	Mouse; *In vivo*; *Ex vivo* (insulin stimulated glucose uptake in skeletal muscle)	*In vivo*: 8 weeks of exposure through diet. Diet: control diet, very high fat diet (VHF), very high fat diet containing elevated amount of POPs through incorporation of whale meat in the diet (VHF_{-POPs}).	Yes	FPG (=); Plasma insulin (=); IPGTT (glucose) (=); IPITT (glucose) (=); Glucose stimulated insulin secretion (insulin) (=); Glucose uptake (muscle) (=)	The metabolic profile of mice fed VHF_{-POPs} was comparable with that of mice of the control group. VHF-fed mice, however, showed increased plasma insulin concentrations, glucose and insulin intolerance (decreased insulin sensitivity), decreased insulin stimulated glucose uptake in skeletal muscle and more ectopic fat accumulation. Pancreatic β-cell function was considered similar in all groups. The authors conclude that mice challenged with VHF_{+POPs} were protected from IR despite elevated POP accumulation in adipose tissue.	WB, P(M)

Source	Chemical	Study	Dose and time	Ins	Endpoints	Observations suggested to be associated with IR	IR
Sun et al. 2009	$PM_{2.5}$	Mouse; *In vivo*; *Ex vivo* (insulin responses in aorta segments)	Mice on a high fat diet were exposed for 128 day to concentrated $PM_{2.5}$ for 6 hours/day (5 days/week).	Yes	FPG (↑); FPI (↑); HOMA-IR (↑); IPGTT (glucose) (↑); Aortic protein expression: pAkt/total Akt (↓), PKC isoforms (↑)	HOMA-IR index was significantly higher for $PM_{2.5}$-exposed mice on a HF diet, compared to HF-diet mice, indicative for whole body IR. Insulin signaling in aortic segments from $PM_{2.5}$-exposed mice was impaired, as illustrated by decreased Akt phosphorylation and changes in PKC expression. Attenuation of the PI3K/Akt insulin signalling pathway was implied.	WB
Xu et al. 2011	$PM_{2.5}$	Mouse; *In vivo*	Mice were exposed to ambient concentrated $PM_{2.5}$ for 6 hours/day (5 days/week) from April 2009 to January 2010. Total: 1,302 h exposure	No	FPG (↑); FPI (=); HOMA-IR (↑); IPGTT (glucose) (↑); Adipose depots (BAT and WAT), liver and muscle protein expression: pAkt/total Akt (↓)	IPGTT showed that long term $PM_{2.5}$-exposure induced IGT. In addition, the HOMA-IR index was significantly increased in exposed mice, thus indicating (systemic) IR. Further testing showed that $PM_{2.5}$-exposure diminished insulin-mediated phosphorylation of Akt at Ser^{473} in the liver, skeletal muscle, and adipose tissues. It was concluded that $PM_{2.5}$-exposure induces systemic and local IR.	WB, P(M), P(A), H
Zheng et al. 2013	$PM_{2.5}$	Mouse; *In vivo*; *In vitro* (human, LX-2 hepatic stellate cell line)	Mice were exposed to concentrated $PM_{2.5}$ at nominal 10x concentrations for 6 hours/day (5 days/week) for a total of 3 to 10 weeks.	No	FPG (↑); FPI (↑); HOMA-IR (↑); IPGTT (glucose) (↑); Liver glycogen content (↓); Liver protein expression: pIRS1 (Ser^{636}) (↑), pIRS1 (Ser^{1101}) (↑), pAkt/total Akt (↓)	Mice exposed for 10 weeks to concentrated $PM_{2.5}$ showed increased FPG and FPI and were insulin resistant as indicated by increased HOMA-IR. Furthermore IGT was observed in IPGTT.<br>In liver, glycogen content was significantly decreased in animals exposed for 10 weeks. Mechanistically, this is supported by increased (inhibitory) phosphorylation of IRS-1 at Ser^{636} and Ser^{1101}. In addition, phosphorylation (activation) of Akt, a key regulator of insulin mediated glycogen metabolism downstream of IRS-1 was decreased. *In vivo* exposure to $PM_{2.5}$ thus reduces insulin signaling via the IRS-1/Akt pathway. This was confirmed *in vitro* in a human stellate cell model. Besides effects on insulin and glucose homeostasis, long term $PM_{2.5}$-exposure also induced expression of inflammatory pathways in liver as well as hepatic steatosis.	WB, H
Brook et al. 2013	$PM_{2.5}$	Human; *In vivo*	Transport of subjects from locations with background levels of $PM_{2.5}$ (5-10 $\mu g/m^3$) to an urban site with high $PM_{2.5}$ levels (mean $11.5 \pm 4.8\ \mu g/m^3$). Exposure occurred during 5 consecutive days, for 4 to 5 h.	No	HOMA-IR (↑)	Sub-acute exposure to prevailing $PM_{2.5}$ concentrations are associated with reduced metabolic insulin sensitivity among healthy adults. Conclusion based on increased HOMA-IR after translocation to more polluted area for 5 days.	WB

Source	Chemical	Study	Dose and time	Ins	Endpoints	Observations suggested to be associated with IR	IR
Xu et al. 2012	$PM_{2.5}$ + Ni	Mouse; *In vivo*	Mice were exposed to a mixture of ambient concentrated $PM_{2.5}$ and nickel ($NiSO_4$) 6 h/day (5 days/week) from September 8 to December 17 2009. Average daily exposure was (mean ± SD): 66.5 ± 44.6 μg/m^3 concentrated $PM_{2.5}$ and 467.9 ± 601.1 ng/ m^3 nickel. (Only results of the combined exposures compared to control are discussed in this table)	No	FPG (↑); FPI (=); HOMA-IR (↑); IPGTT (glucose) (=)	Although no change in glucose tolerance and FPI was observed, FPG and HOMA-IR were significantly increased in mice exposed to a mixture of concentrated $PM_{2.5}$ and nickel compared to the control condition, and synergistically increased compared to $PM_{2.5}$ alone. Increased HOMA-IR is indicative for induction of IR.	WB
Hoppe and Carey 2007	Polybrominated diphenyl ethers (PBDE)	Mouse; *Ex vivo*; *In vitro*	14 mg KD; 2 or 4 weeks; Isolated adipocytes: 30 μg/mL 90 min.	Yes	Glucose oxidation (↓)	Isolated adipocytes from mice exposed for 4 weeks to 14 mg KD PBDE showed decreased insulin stimulated glucose oxidation. No effect was observed for mice exposed for 2 weeks, nor for isolated adipocytes from control mice, exposed *in vitro* to PBDEs. Reduction in insulin signaling is a proposed explanation for the observed effects. The decreased glucose oxidation rate and significant increase in lipolysis are suggested to be associated with IR.	P(A)
Hsu et al. 2010	2,3,7,8-Tetrachlorodibenzo-p-dioxin (TCDD)	Mouse; *In vitro* (3T3-L1 cell line)	0.1, 1 and 10 nM; 3 d<br>0.1, 1 and 10 nM; 20 min. (during glucose uptake assay);	Yes	Glucose uptake (3T3-L1) (↓)	Pretreatment of cells with TCDD did not alter insulin responsiveness with regard to glucose uptake. Insulin stimulated glucose uptake in the presence of TCDD, however, was dose-dependently attenuated. Inhibition of AhR gave comparable results, indicating AhR-independent action of TCDD. The authors postulate that "the involvement of TCDD in interfering with glucose uptake by adipocytes may lead to IR and disruption of glucose homeostasis."	P(A)

Source	Chemical	Study	Dose and time	Ins	Endpoints	Observations suggested to be associated with IR	IR
Nishiumi et al. 2010	2,3,7,8-Tetrachlorodibenzo-p-dioxin (TCDD)	Mouse; *In vitro* (3T3-L1 cell line)	1, 5 or 10 nM; 1 h, 24 h or 48 h	Yes	Adipocyte (3T3-L1) gene expression: IRecβ (↓), IRS-1 (↓), GLUT4 (↓); Adipocyte (3T3-L1) protein expression: IRecβ (↓), IRS-1 (↓), GLUT4 (↓), ERK 1/2 (=), JNK (=), p38 MAPK (=), pERK 1/2 (↑), pJNK (↑), pp38 MAPK (=); Glucose uptake (3T3-L1) (↓)	Long term (24 h and 48 h) TCDD treatment affected the insulin signaling pathway in 3T3-L1 adipocytes demonstrated by decreased mRNA and protein expression of IRecβ, IRS-1 and GLUT4. Furthermore, insulin stimulated glucose uptake was completely absent in cells treated with 10 nM TCDD for 48 h, illustrative for TCDD triggering IR. Entanglement of the underlying pathway revealed that part of the effect could be explained by TCDD-induced (via ERK1/2, JNK and AhR activation) TNF-α secretion, which caused decreased expression of IRecβ, IRS-1 and GLUT4 in an autocrine fashion (via TNFR1 and NFκB). Downregulation of components of the insulin signaling cascade was also related to AhR activation. All these results support induction of IR in a *in vitro* adipocyte model by long term TCDD exposure.	P(A)
Sargis et al. 2012	Tolylfluanid	Rat, mouse, human; *In vitro* (Primary adipocyte; 3T3-L1 cell line)	Variable, but main effects: 100 nM, 48 h	Yes	Adipocyte gene expression: IRS-1 (↓); Adipocyte protein expression: pAkt/total Akt (↓), IRecβ (=), pIRecβ (=), P85-PI3K (=), IRS-1 (↓), pIRS-1 (↓), PI3K-IRS-1 (↓)	Sargist et al. (2012) showed that tolyfluanid induces cellular IR in primary murine and human adipocytes by disrupting the insulin signaling cascade. Main target is suggested to be the PI3K/Akt pathway, and tolyfluanid is suggested to attenuate insulin stimulated Akt phosphorylation by reducing IRS-1 levels in adipocytes.	P(A)

Abbreviations: AhR, Aryl hydrocarbon receptor; Erk, extracellular signal-regulated kinase; FPG, fasting plasma glucose; FPI, fasting plasma insulin; G6Pc, glucose-6-phosphatase catalytic subunit; GIR, glucose infusion rate; GLUT4, glucose transporter 4; GSK3, glycogen synthase kinase 3; GTT, glucose tolerance test; HGP, hepatic glucose production; HOMA-IR, homeostasis model assessment-insulin resistance; IGT, impaired glucose tolerance; IPGTT, intraperitoneal glucose tolerance test; IPITT, intraperitoneal insulin tolerance test; IRec, insulin receptor; IRS-1, insulin receptor substrate 1; ITT, insulin tolerance test; IVGTT, intravenous glucose tolerance test; JNK, c-Jun N-terminal kinase; KD, kg^{-1} day^{-1}; MAPK, mitogen-activated protein kinase; NFκB, nuclear factor κB; OGTT, oral glucose tolerance test; PEPCK, phosphoenolpyruvate carboxykinase; PI3K, phosphatidylinositol 3-kinase; PKC, protein kinase C; PM, particulate matter; pX, phosphorylated X; Rd, glucose disappearance rate; ROS, reactive oxygen species; SREBP, sterol regulatory element-binding protein; TNF-α, tumor necrosis factor-α; TNFR1, TNF receptor 1.

[a]Columns give information on the compound studied, the species used and type of experiments (*in vivo*, *ex vivo* or *in vitro*) (Study), the dose and length of exposure (Dose and time), whether insulin was present in some or all of the assays used to assess the degree of IR (Ins), the endpoints

tested in each of the reported studies and their accompanying results (↑ = increased; ↓ = decreased; = = unaltered), a description of the observations which made authors decide on presence of IR after pollutant exposure and finally, the type of IR studied (WB = whole body; H = hepatic; P = peripheral (A = adipose tissue; M = muscle)).

[b]Only the general trend is provided for pollutant effects. In some of the cases, co-treatment with different diet types may influence the results on insulin sensitivity related endpoints or not. For those studies, only the general results of the pollutant-treatments are given. Furthermore, if more doses were tested, compiled results which made the authors decide on the presence of IR are represented. Studies are sorted alphabetically based on the name of the chemical.

[c]Only changes of HFC or VHF/S diet with regard to insulin/glucose homeostasis are given compared to control diets. Comparison with HFR or VHF/S_{-POPs} sometimes deviates from the observations reported here. For all results we refer to the original papers (Ibrahim et al. 2011; Ruzzin et al. 2010).

References

Alonso-Magdalena P, Morimoto S, Ripoll C, Fuentes E, Nadal A. 2006. The estrogenic effect of bisphenol A disrupts pancreatic beta-cell function in vivo and induces insulin resistance. Environ Health Perspect 114:106-112.

Barnes DM, Kircher EA. 2005. Effects of mercuric chloride on glucose transport in 3T3-L1 adipocytes. Toxicol In Vitro 19:207-214.

Batista TM, Alonso-Magdalena P, Vieira E, Amaral ME, Cederroth CR, Nef S, et al. 2012. Short-term treatment with bisphenol-a leads to metabolic abnormalities in adult male mice. Plos One 7:e33814.

Brook RD, Xu X, Bard RL, Dvonch JT, Morishita M, Kaciroti N, et al. 2013. Reduced metabolic insulin sensitivity following sub-acute exposures to low levels of ambient fine particulate matter air pollution. Sci Total Environ 448:66-71.

Fang X, Gao G, Xue H, Zhang X, Wang H. 2012. Exposure of perfluorononanoic acid suppresses the hepatic insulin signal pathway and increases serum glucose in rats. Toxicology 294:109-115.

Hoppe AA, Carey GB. 2007. Polybrominated diphenyl ethers as endocrine disruptors of adipocyte metabolism. Obesity 15:2942-2950.

Hsu HF, Tsou TC, Chao HR, Kuo YT, Tsai FY, Yeh SC. 2010. Effects of 2,3,7,8-tetrachlorodibenzo-p-dioxin on adipogenic differentiation and insulin-induced glucose uptake in 3T3-L1 cells. J Hazard Mater 182:649-655.

Ibrahim MM, Fjære E, Lock EJ, Froyland L, Jessen N, Lund S, et al. 2012. Metabolic impacts of high dietary exposure to persistent organic pollutants in mice. Toxicol Lett 215:8-15.

Ibrahim MM, Fjære E, Lock EJ, Naville D, Amlund H, Meugnier E, et al. 2011. Chronic consumption of farmed salmon containing persistent organic pollutants causes insulin resistance and obesity in mice. Plos One 6:e25170.

Jubendradass R, D'Cruz S, Mathur P. 2012. Long-term exposure to nonylphenol affects insulin signaling in the liver of adult male rats. Hum Exp Toxicol 31:868-876.

Khalil A, Villard PH, Dao MA, Burcelin R, Champion S, Fouchier F, et al. 2010 Polycyclic aromatic hydrocarbons potentiate high-fat diet effects on intestinal inflammation. Toxicol Lett 196:161-167.

Lim S, Ahn SY, Song IC, Chung MH, Jang HC, Park KS, et al. 2009. Chronic exposure to the herbicide, atrazine, causes mitochondrial dysfunction and insulin resistance. Plos One 4:e5186.

Mostafalou S, Eghbal MA, Nili-Ahmadabadi A, Baeeri M, Abdollahi M. 2012. Biochemical evidence on the potential role of organophosphates in hepatic glucose metabolism toward insulin resistance through inflammatory signaling and free radical pathways. Toxicol Ind Health 28:840-851.

Nishiumi S, Yoshida M, Azuma T, Yoshida Ki, Ashida H. 2010. 2,3,7,8-Tetrachlorodibenzo-p-dioxin impairs an insulin signaling pathway through the induction of tumor necrosis factor-alpha in adipocytes. Toxicol Sci 115:482-491.

Palacios J, Roman D, Cifuentes F. 2012. Exposure to low level of arsenic and lead in drinking water from Antofagasta city induces gender differences in glucose homeostasis in rats. Biol Trace Elem Res 148:224-231.

Rajesh P, Sathish S, Srinivasan C, Selvaraj J, Balasubramanian K. 2013. Diethyl Hexyl Phthalate (DEHP) is associated with insulin resistance in adipose tissue of male rat: Protective role of antioxidant vitamins (C & E). J Cell Biochem 114:558-569.

Ruzzin J, Petersen R, Meugnier E, Madsen L, Lock EJ, Lillefosse H, et al. 2010. Persistent organic pollutant exposure leads to insulin resistance syndrome. Environ Health Perspect 118:465-471.

Sargis RM, Neel BA, Brock CO, Lin Y, Hickey AT, Carlton DA, et al. 2012. The novel endocrine disruptor tolylfluanid impairs insulin signaling in primary rodent and human adipocytes through a reduction in insulin receptor substrate-1 levels. Biochim Biophys Acta 1822:952-960.

Srinivasan C, Khan AI, Balaji V, Selvaraj J, Balasubramanian K. 2011. Diethyl hexyl phthalate-induced changes in insulin signaling molecules and the protective role of antioxidant vitamins in gastrocnemius muscle of adult male rat. Toxicol Appl Pharmacol 257:155-164.

Sun Q, Yue P, Deiuliis JA, Lumeng CN, Kampfrath T, Mikolaj MB, et al. 2009. Ambient air pollution exaggerates adipose inflammation and insulin resistance in a mouse model of diet-induced obesity. Circulation 119:538-U91.

Xu X, Liu C, Xu Z, Tzan K, Zhong M, Wang A, et al. 2011. Long-term exposure to ambient fine particulate pollution induces insulin resistance and mitochondrial alteration in adipose tissue. Toxicol Sci 124:88-98.

Xu X, Rao X, Wang TY, Jiang S, Ying Z, Liu C, et al. 2012. Effect of co-exposure to nickel and particulate matter on insulin resistance and mitochondrial dysfunction in a mouse model. Part Fibre Toxicol 9:40.

Zheng Z, Xu X, Zhang X, Wang A, Zhang C, Huettemann M, et al. 2013. Exposure to ambient particulate matter induces a NASH-like phenotype and impairs hepatic glucose metabolism in an animal model. J Hepatol 58:148-154.
